# A Disposable Dopamine Sensor Based on Oxidized Cellulose Nanofibril-Modified SPCE

**DOI:** 10.3390/mi16070743

**Published:** 2025-06-25

**Authors:** Feriel Boussema, Sondes Bourigua, Zayneb Jebali, Hatem Majdoub, Nicole Jaffrezic-Renault, Hamdi Ben Halima

**Affiliations:** 1 Laboratory of Interfaces and Advanced Materials, Faculty of Sciences, University of Monastir, Monastir 5000, Tunisia; ferielbousema@hotmail.com (F.B.); bouriguasondes@yahoo.fr (S.B.); zayneb90@hotmail.fr (Z.J.); 2 Department of Chemistry, College of Science, Qassim University, Buraidah 51452, Saudi Arabia; hatemmajdoub.fsm@gmail.com; 3 UTINAM Institute, Marie and Louis Pasteur University, 25010 Besançon, France; nicole.jaffrezic-renault@univ-fcomte.fr

**Keywords:** TEMPO-oxidized cellulose nanofibrils, TOCNF-modified SPCE, electrochemical detection, dopamine

## Abstract

Dopamine is a major catecholamine neurotransmitter that plays an essential role in the functioning of the human central nervous system. Imbalances in dopamine levels are associated with neurological disorders and depression. Thus, measuring the concentration of DA in human body fluids is significantly important. In this work, TEMPO-oxidized cellulose nanofibrils (TOCNFs) extracted from marram grass (*Ammophilia arenaria*), harvested in the central western part of Tunisia, were utilized to modify disposable screen-printed carbon electrodes (SPCEs) for the sensitive detection of dopamine in biological fluids. Differential pulse voltammetry (DPV) measurements displayed a sensitivity of 7.92 µA/µM and a detection limit of 10 nM. The disposable TOCNF-modified SPCE presents a charge transfer coefficient, α, comparable to that of a TOCNF/graphene/AgNP composite-modified GCE. Moreover, it exhibits good repeatability (RSD = 1.9%), good reproducibility (RSD = 2.3%), and appreciable storage stability (91% of its initial response after 3 weeks). The prepared disposable sensor showed satisfactory recovery of dopamine in human urine samples.

## 1. Introduction

Dopamine (DA), a monoamine neurotransmitter, is among the most extensively studied due to its vital role in human physiology [1]. DA controls certain physiological conditions such as attention, mood, memory, learning, movement, behavior, and mental cognition [2,3]. Disruptions in dopamine (DA) levels in the brain are associated with a range of neurological and psychiatric malfunctions, including depression, addiction, schizophrenia, and neurodegenerative diseases like Alzheimer’s and Parkinson’s [4]. Dopamine typically exists in blood serum, and its level is very low, in the range of 0.01 to 1.0 µmol/L [5]. Therefore, detecting and quantifying dopamine is essential for understanding these brain disorders. This is why a straightforward, selective, and sensitive method for detecting dopamine is necessary for monitoring its levels in the body. Different techniques have been used for the detection of DA, such as chromatography [6,7], fluorescence [8], chemiluminescence [9], and electrochemistry [10,11,12,13]. Of these techniques, the electrochemical process attracted the most attraction due to its wide selectivity, high sensitivity, simple instrumentation with a rapid response, and low cost. Dopamine is a type of catecholamine (pKa 8.9) that can be detected electrochemically when it is oxidized on an electrode surface [14]. The detection sensitivity can be adjusted by modifying an electrode with appropriate materials, such as SAMs [15], polymers [16,17,18], metal nanoparticle arrays [19,20], metal oxides [21,22], and carbon materials [23,24]. Recently, the use of renewable resources has become increasingly important since they are eco-friendly. Cellulose nanofibrils (CNFs) are natural nanomaterials extracted from plant cell walls. With their diameter size of a few nanometers, CNFs present desirable properties such as exceptional strength, excellent stiffness, high surface area, and high surface concentration of hydroxyl groups, which are easily accessible for surface modification. As biodegradable nanomaterials, CNFs are highly attractive for a variety of applications, including nanocomposite materials, surface-modified materials, and transparent paper with specialized functions [25]. Mechanical methods are employed to extract them from cellulose fibrils [26,27,28]. In this study, CNFs were mechanically extracted from marram grass (*Ammophilia arenaria*) and oxidized by 2,2,6,6 tetramethylpiperidine-1-oxyl (TEMPO) to generate carboxyl groups, leading to the creation of TOCNFs. Commercial screen-printed carbon electrodes (SPCEs) were modified with TOCNFs for the sensitive detection of dopamine in biological samples. The use of disposable SPCEs is of high interest for testing biological samples [29,30]. The high affinity of sulfated or carboxylated CNFs towards carbonaceous materials (MWCNTs, graphene, tetrahedral amorphous carbon, and glassy carbon) was shown in the preparation of composites and modified electrodes [31,32,33,34]. An aqueous suspension of TOCNFs is simply deposited on SPCEs by drop-casting.

## 2. Materials and Methods

### 2.1. Chemicals and Reagents

K_4_[Fe(CN)_6_]·3H_2_O, K_3_[Fe(CN)_6_], KCl, dopamine, and serotonin were purchased from Fluka Chemika. NaOH, KOH, NaClO_2_, NaClO, H_3_BO_3_, ethanol, and 2,2,6,6 tetramethylpiperidine-1-oxyl (TEMPO) were purchased from Sigma-Aldrich. The buffer solution used for all experiments was phosphate-buffered saline (PBS) containing 137 mM NaCl, 2.7 mM KCl, 0.01 M KH_2_PO_4_, and 0.01 M K_2_HPO_4_, pH 7. Ultrapure water (18 MΩ·cm resistance), obtained from a Millipore Milli-Q system, was used to prepare all solutions. The cellulose nanofibrils were isolated from marram grass (*Ammophila arenaria*) collected in the central western part of Tunisia (Gafsa).

### 2.2. Apparatus

Electrochemical measurements were carried out using a Mini Potentiostat (DY2100 Series) from Digi-Ivy, Inc. (Austin, TX, USA). Screen-printed carbon electrodes (SPCEs) were purchased from DropSens, Llanera, Spain. The SPCE platform comprised a 4 mm diameter carbon working electrode, a silver pseudo-reference electrode, and a carbon counter electrode. DPV was used to investigate the electrochemical behavior of dopamine under the following conditions: a scan rate of 50 mV.s^−1^ after an accumulation time of 20 s, an initial potential of −0.2 V, a final potential of +0.8 V, a pulse width of 500 ms, a pulse period of 500 ms, and a modulation amplitude of 50 mV.

The infrared (IR) spectrum of the TOCNF sample was obtained using an FTIR spectrophotometer (BX FTIR system spectrometer, Perkin Elmer Company, Waltham, MA, USA) in the absorbance mode from 4000 to 400 cm^−1^. The TOCNF sample was incorporated into potassium bromide (KBr) powder and pressed into a 1 mm pellet.

The TEM image of the TONFCs was captured using a JEOL 200CX transmission electron microscope (JEOL Ltd., Tokyo, Japan) at 80 kV. A 0.5 µL diluted suspension of TOCNFs (about 0.1%) was placed onto a 300-mesh carbon-coated grid.

### 2.3. Production of TOCNFs

Marram grass (*Ammophila arenaria*) was utilized as the biomass for CNF production. Before lignin extraction, the stems were ground using a cutting mill (Cutting Mill SM 100 from Retsch, Retsch GmbH, Haan Germany). The ground stems were delignified in a 2 wt% NaOH solution under heating at 70–80 °C for 2 h, with mechanical stirring. The fibers were bleached twice with NaClO_2_ to eliminate the remaining lignin. Cellulose fibers were obtained from the bleached pulp after removing hemicellulose through a treatment with a 10% KOH solution containing 1% H_3_BO_3_ for 10 h at room temperature. Cellulose fibers were extracted and subsequently oxidized to introduce carboxyl groups. The oxidation was performed at pH 10 according to the method described earlier [28]. In total, 5 g of cellulose fibers were initially dispersed in 500 mL of 0.05 M sodium phosphate buffer (pH 7) containing 25 mg of TEMPO and 250 mg of NaBr. Sodium chlorite (80%, 1.13 g, 10 mmol) and a 2 M sodium hypochlorite solution (0.5 mL, 1.0 mmol) were added simultaneously to the flask. The suspension was magnetically stirred at 60 °C for 6 h. Throughout the oxidation process, the modified pulp turned yellow as a result of free chlorine generation. The oxidation was halted by adding 100 mL of ethanol, after which the oxidized fibers were filtered and washed multiple times with water to remove the salt. Then, the cellulose fiber suspension was disintegrated using a high-pressure homogenizer (NS1001L PANDA 2K, GEA Mechanical Equipment, Parma, Italy). The homogenization process was carried out in two steps. Initially, the aqueous fiber suspension (1.5 wt%) was passed through the homogenizer three times at a pressure of 300 bar (4350 psi) until it took on a gel-like consistency. Subsequently, fibrillation was continued with five additional passes at a pressure of 600 bar (8700 psi) [28,34].

### 2.4. TOCNF-Modification of the Working Electrode

In total, 7 µL of an aqueous TOCNF suspension (1 mg/mL) was deposited on the working SPCE. The modified surface was left to dry at ambient temperature overnight. Good interfacial bonding of the TOCNF film on the SPCE was obtained due to its high affinity for carbonaceous surfaces [31,32,33,34].

## 3. Results and Discussion

### 3.1. Morphological Characterization of TOCNFs

**Figure 1:**
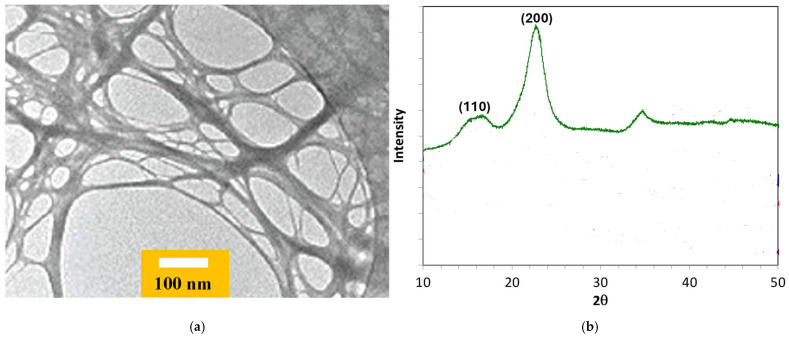
(**a**) TEM observation of TOCNFs; (**b**) XRD spectrum of TOCNFs.

Figure 1a presents a TEM observation of cellulose nanofibrils. Their width is within the range of 5–6 nm. X-ray diffraction (XRD) analysis confirmed the crystalline structure of the TOCNFs (Figure 1b); the TOCNFs exhibit a weak peak at 14°, assigned to the [110] lattice plane, and a sharper peak at 22°, corresponding to the [200] lattice plane of cellulose [35].

### 3.2. FTIR Characterization of TOCNFs

**Figure 2:**
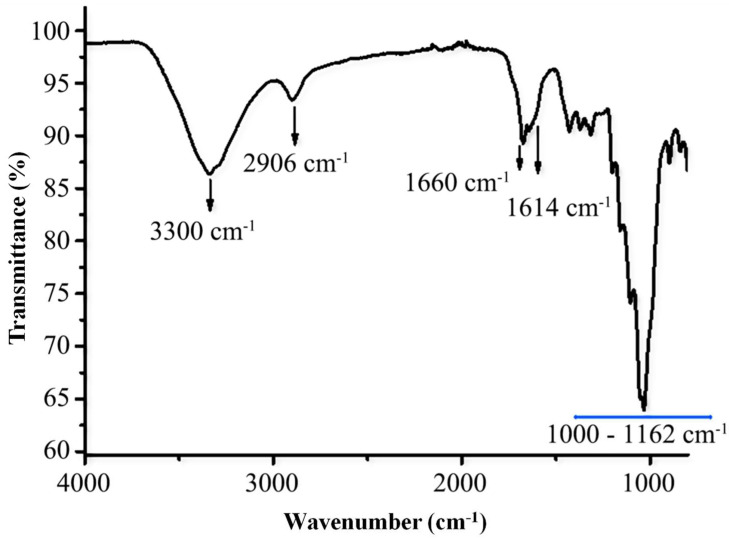
Fourier transform infrared spectroscopy (FTIR) spectrum of TOCNFs.

The FTIR spectrum of the TOCNFs, presented in Figure 2, displays distinct absorption bands of polysaccharides. An intense and broad band centered around 3300 cm^−1^ corresponds to O–H stretching vibrations, indicative of hydroxyl groups. Likewise, the band at 2906 cm^−1^ is assigned to the stretching vibration of the (C–H) bond.

Furthermore, the band at 1660 cm^−1^ is attributed to the O–H bending vibration of adsorbed water. The band at 1614 cm^−1^ corresponds to the carboxyl groups formed following TEMPO-mediated oxidation of the primary alcohol groups on the CNF surface. The characteristic absorption bands of the glucosidic ring are observed within the 1000–1162 cm^−1^ region.

### 3.3. Electrochemical Characterization of TOCNF-Modified SPCE

**Figure 3:**
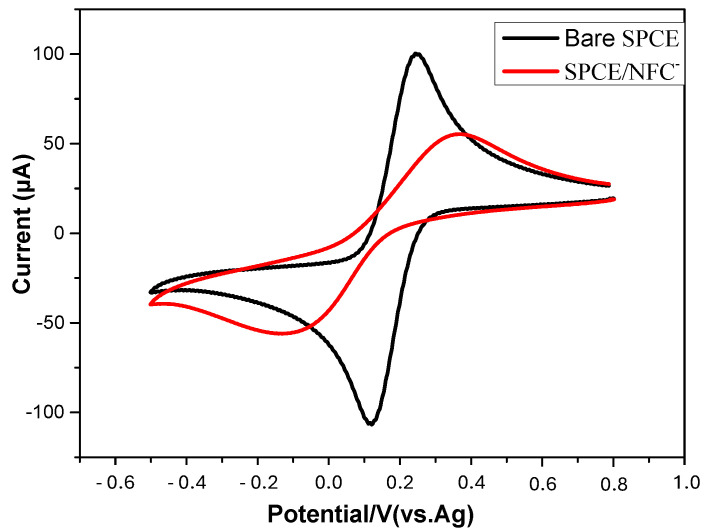
Cyclic voltammograms of the bare SPCE and the TOCNF-modified SPCE in 0.1 M KCl solution containing 5 mM [Fe(CN)_6_]^3−/4−^ at a scan rate of 50 mV.s^−1^.

Cyclic voltammetry was performed to investigate the electrode’s behavior after the deposition of the TOCNF film on the SPCE surface. Figure 3 illustrates the change in electroactivity of the [Fe(CN)_6_]^3−/4−^ redox probe on both the bare and TOCNF-modified SPCEs. The cyclic voltammogram displays a quasi-reversible redox peak, with a ΔEp of 100 mV and a current ratio of approximately 1:1 (Ia = 100 µA; Ic = 108 µA) between the anodic and cathodic peaks, indicating that the SPCE undergoes an interfacial reaction with the [Fe(CN)_6_]^3−/4−^ couple. Following the deposition of the TOCNFs, ΔEp is equal to 490 mV, and the peak currents decrease (Ia = 56 µA; Ic = 58 µA). The redox system becomes irreversible; this is due to the poor conductivity of the TOCNFs and their negative charge, which pushes back the negatively charged redox system. This result confirms the stability of the TOCNF film on the SPCE surface during the electrochemical measurements.

### 3.4. Electrochemical Behavior of Dopamine

**Figure 4:**
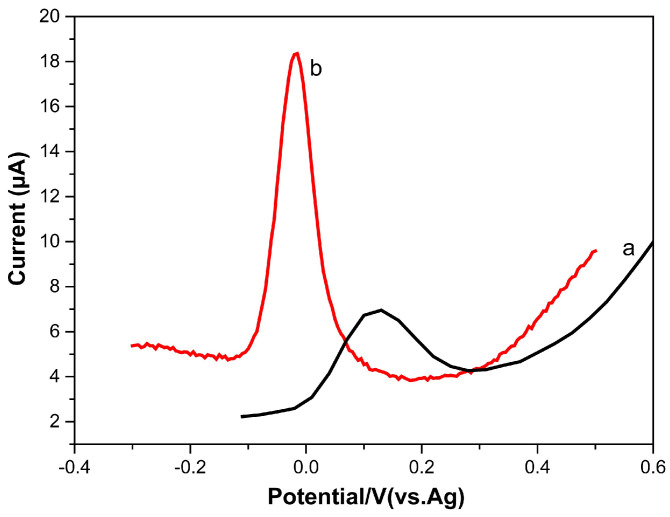
DPV of 10 µM of dopamine on the bare SPCE (a) and TOCNF-modified SPCE (b). Experimental conditions: 0.01 M phosphate-buffered solution (pH 7.0), scan rate 50 mV.s^−1^.

The electrochemical performance of 10 µM dopamine on the bare SPCE (a) and on the TOCNF-modified SPCE (b) was investigated by DPV in PBS (pH 7.0) (Figure 4). The oxidation peak potential was found to be 0.11 V for dopamine at the bare SPCE and 0.03 V at the TOCNF-modified SPCE. Additionally, the intensity increased by a factor of 4.9 following the modification of the SPCE. This intensity amplification is attributed to the enhanced adsorption of dopamine molecules on the TOCNF film at pH 7. This irreversible phenomenon was observed with the inner sphere cationic dopamine on sulfated CNFs [30], which shows that dopamine molecules have specific chemical interactions with the negatively charged CNF surface; besides electrostatic interactions, hydrogen bonds can be formed between the hydroxyl groups of dopamine and TOCNFs.

#### 3.4.1. Effect of the Modified Amount of TOCNFs

**Figure 5:**
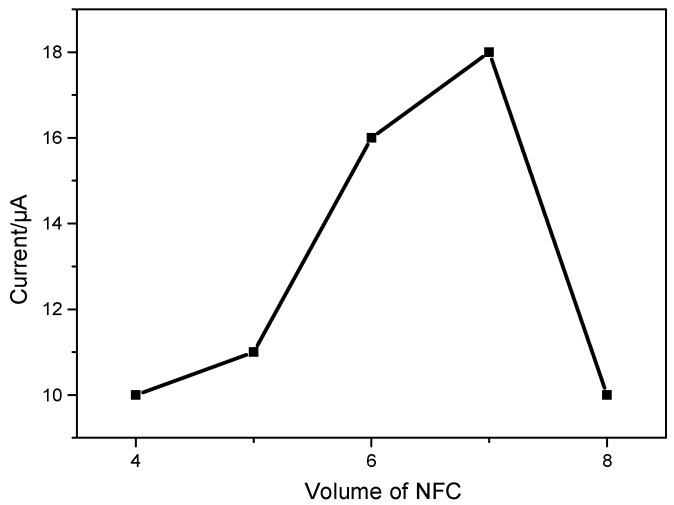
The effect of the volume of a TOCNF suspension on the DA peak current. Experimental conditions: 0.01 M phosphate-buffered solution (pH 7.0), scan rate 50 mV.s^−1^.

The impact of the amount of TOCNFs on the dopamine peak current was investigated. The peak current increased rapidly when the volume of deposited TOCNF suspension (1 mg/mL) increased from 4 to 7 µL (Figure 5). However, when the amount of TOCNFs exceeded 7 µL, the peak current dramatically decreased. The results might be ascribed to the thicker film of TOCNFs, which hindered the electrical transfer to the surface of the electrode. Therefore, a TOCNF suspension of 7 µL was utilized to modify the SPCE.

#### 3.4.2. Influence of pH

**Figure 6:**
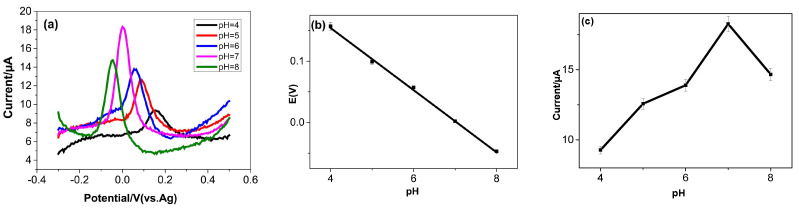
Current vs. potential at different pH (**a**), effect of pH on peak potential (**b**), and effect of pH on peak current (**c**) of 0.1 mM of dopamine at TOCNF-modified SPCE (0.01 M phosphate-buffered solution (pH 7.0)).

The DPV peak potentials and DPV peak currents of dopamine oxidation were observed within a pH range of 4.0 to 8.0 (Figure 6a). The peak potential of DA, as a function of the solution pH (Figure 6b), varies according to the following linear equation:

Epa (DA) = −0.050pH + 0.001 (R^2^ = 0.997) (1)

The slope of 50 mV pH^−1^, which is in close agreement with the Nernst value (59 mV/pH at 25 °C), supports the participation of an equivalent number of protons and electrons in the dopamine redox reaction. This finding is consistent with the previously established mechanism of dopamine oxidation [36].

Figure 6c illustrates that the anodic peak current (Ipa) of dopamine increased progressively with pH up to 7; this was followed by a decrease at higher pH values. At pH values higher than 7, the interaction between dopamine molecules and TOCNFs diminishes. A pH of 7.0, corresponding to the pH of physiological fluids, was selected for all subsequent experiments in this study.

#### 3.4.3. Effect of Scan Rate

**Figure 7:**
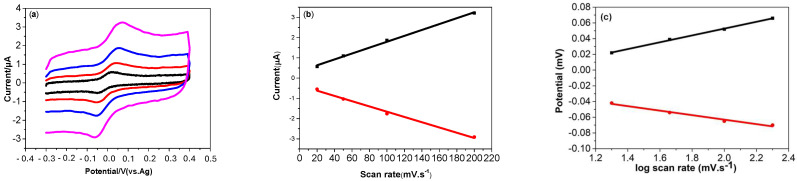
Scan rate analysis: (**a**) overlay of cyclic voltammogram for oxidation of dopamine at different scan rates (black 20 mV/s; red 50 mV.s^−1^, blue 100 mV.s^−1^, pink 200 mV.s^−1^), (**b**) plot of peak current vs. scan rate, (**c**) plot of peak potential vs. logarithm of scan rate. Conditions: TOCNF-modified SPCE (0.01 M phosphate-buffered solution (pH 7.0)).

To investigate the electrochemical kinetics at the TOCNF-modified SPCE, cyclic voltammograms (CVs) of a 0.5 µM DA solution were recorded at varying scan rates (20 to 200 mV.s^−1^). As shown in Figure 7a, both the anodic and cathodic peak currents exhibited a linear increase with the scan rate. Additionally, as the scan rate was increased, the anodic and cathodic peak potentials shifted towards more positive and negative values, respectively, indicating a quasi-reversible reaction for dopamine.

Moreover, the redox peak current (Ipa and Ipc) of DA showed good linear relationships as a function of the scan rate, with good correlation coefficients (R^2^), as shown in Figure 7b.

Ipa (μA) = 0.014 (mV.s^−1^) + 5.5 × 10^−5^ (R^2^ = 0.998) (2)

Ipc (μA) = −0.012 (mV.s^−1^) + 6.9 × 10^−4^ (R^2^ = 0.998) (3)

The linear dependence of the redox peak current upon the scan rate, in the range 20 mV.s^−1^ to 200 mV.s^−1^, demonstrates an adsorption-controlled process [37]. Also, plots of the logarithm of the peak current versus the logarithm of the scan rate gave straight lines with slopes of 0.84 and 0.83 for the anodic and cathodic currents, respectively. The values of the slopes are close to the theoretical value of 1.0, which is expected for an ideal adsorption-controlled electrochemical reaction.

**Figure 8:**
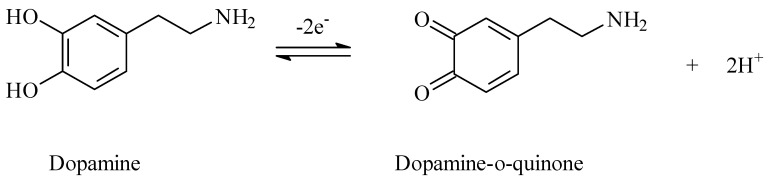
Mechanism of dopamine electrochemical oxidation.

The electron number involved in the oxidation of dopamine was calculated based on the following equation:

Ipa = nFQv/4RT(4)

where Ipa represents the anodic peak current, n is the number of electrons transferred, Q is the amount of charge integrated from the area of voltammetric peak, F is the Faraday constant (96500 C/mol), T is the temperature in Kelvin (298 K), and R is the universal gas constant (8.314 J.mol^−1^) [33]. From the linear relationship between I_p_ and the scan rate (v), the value of n was calculated to be 2. It follows that the oxidation of dopamine involves the transfer of two electrons and two protons. Taking into account both the experimental results and the existing literature, the proposed mechanism for dopamine oxidation on the TOCNF-modified SPCE is illustrated in Figure 8.

Also, Figure 7c demonstrates that both the Epa and Epc of DA are linearly proportional to log ν, as shown in Equations (5) and (6):

Epa (mV) = 0.043 logv + 0.001 (R^2^ = 0.998) (5)

Epc (mV) = −0.028 logv + 9.38 × 10^−4^ (R^2^ = 0.998) (6)

According to Laviron’s equation [38], for DA, the linear relationship between Epa and log ν presents a slope of (2.3 RT/αnF) equal to 0.043, and the slope of Epc versus log v is (2.3 RT/(1 − α)nF) equal to −0.028. From this, the value of αn is calculated to be 0.60, and the charge transfer coefficient α is determined to be 0.30. This value found on the disposable TOCNF-modified SPCE is close to that found on the TOCNF/graphene/AgNP composite-modified GCE, 0.39 [33]. This point shows that, for a simple disposable system, i.e., the TOCNF-modified SPCE, the charge transfer coefficient value is close to that of a sophisticated system, i.e., the TOCNF/graphene/AgNP composite-modified GCE.

### 3.5. Analytical Performance of the Dopamine Sensor

The differential pulse voltammetry (DPV) technique provides higher sensitivity and better resolution compared to cyclic voltammetry (CV), as it enables the direct cancellation of the capacitive current. Thus, DPV was employed to assess the analytical performance of the TOCNF-modified SPCE for the quantitative determination of dopamine (DA) in 0.1 M PBS at pH 7, using a scan rate of 50 mV.s^−1^.

**Figure 9:**
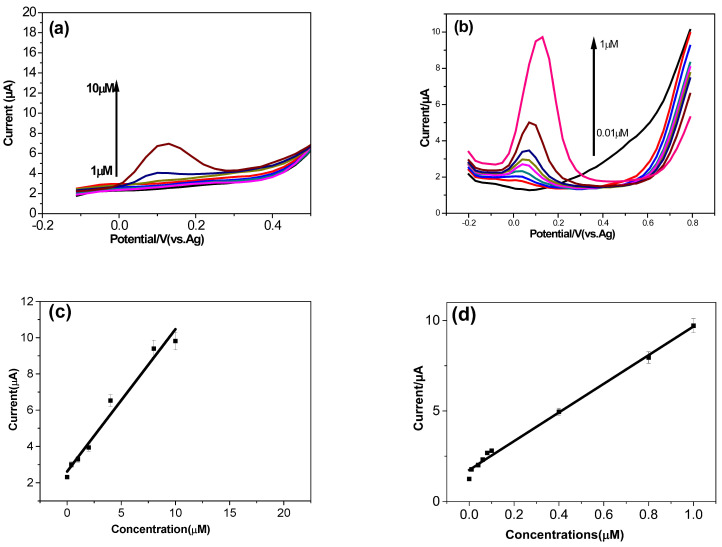
DPV detection of dopamine in a concentration range from 1 to 10 µM on the bare SPCE (**a**) and from 0.01 µM to 1 µM on the TOCNF-modified SPCE (red 0.01 µM, blue 0.04 µM, green 0.06 µM, pink 0.08 µM, khaki 0.1 µM, black 0.4 µM, brown 0.8 µM, pink 1 µM) (0.01 M phosphate-buffered solution (pH 7.0)) (**b**) in 0.01 M phosphate-buffered solution (pH 7.0); scan rate 50 mV/s. Calibration curve of dopamine at the bare SPCE (**c**) and at the TOCNF-modified SPCE (0.01 M phosphate-buffered solution (pH 7.0)) (**d**). Experimental conditions: 0.01 M phosphate-buffered solution (pH 7.0), scan rate 50 mV/s.

Figure 9a presents the DPV signals of dopamine (DA) with increasing concentrations from 1 to 10 μM at the bare screen-printed carbon electrode (SPCE). The oxidation peaks of DA gradually increased with the rise in DA concentration. Figure 9b displays the DPV signals for varying concentrations of dopamine at the SPCE. It was observed that the anodic peak current increased with the dopamine concentration, ranging from 0.01 to 1 μM.

Figure 9c,d show a good linear relationship between the current and the concentration of dopamine on the bare SPCE and the TOCNF-modified SPCE, respectively. The sensitivity for the bare SPCE was measured as 0.78 µA.µM^−1^. The sensitivity for the TOCNF-modified SPCE is 7.92 µA.µM^−1^, and a detection limit of 0.01µM was obtained. For higher concentrations of dopamine, the sensitivity decreases to 1.4 µA.µM^−1^. The dynamic range is 1 µM to 100 µM.

In Table 1, the analytical performance of the disposable TOCNF-modified SPCE is compared to that of previously published CNF-based electrochemical sensors. The TOCNF-modified SPCE presents a lower detection limit than that of the sulfated CNF/MWCNT composite-modified taC electrode [30]. When AgNP was added to the TOCNF/graphene composite for GCE modification [32], a lower detection limit than that of the TOCNF-modified SPCE was obtained. Nevertheless, without AgNP, the modified GCE electrode presents a lower transfer coefficient (0.014 s^−1^) than that of the TOCNF-modified SPCE (0.39 s^−1^).

The stability of the TOCNF-modified SPCE was assessed by monitoring the oxidation current of 0.5 µM dopamine in 0.1 M PBS at pH 7 after storage at 4 °C. No significant decrease in current was observed during the first week (RSD = 1.9%), and the response remained stable at 91% of its initial value after three weeks.

The inter-sensor reproducibility of the TOCNF-modified SPCE for dopamine detection was evaluated using four modified SPCEs. The relative standard deviation (RSD) was found to be 2.3% (n = 4).

### 3.6. Detection of Dopamine in the Presence of Uric Acid and Ascorbic Acid and Another Neurotransmitter, Serotonin

**Figure 10:**
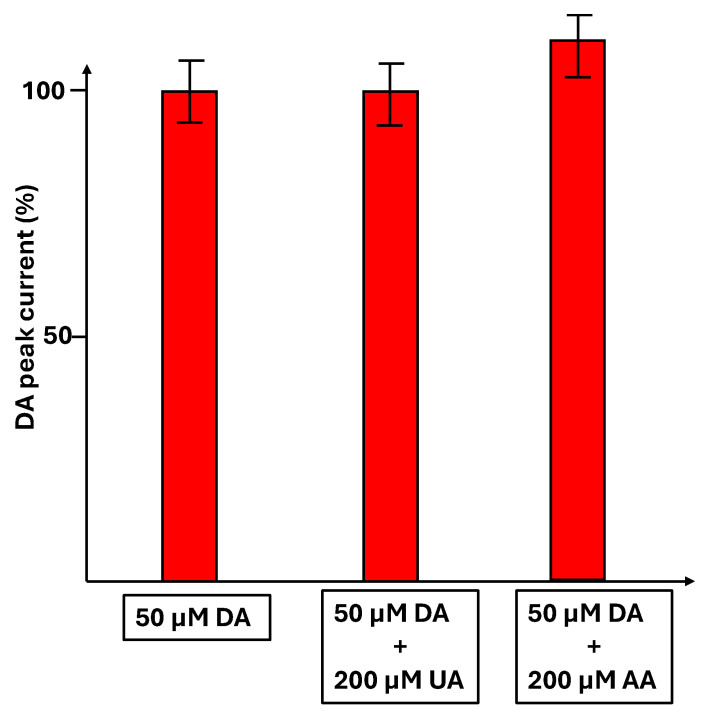
Percentage of DA peak current (50 µM DA) in the absence and the presence of uric acid (200 µM) and ascorbic acid (200 µM).

The detection of dopamine (50 µM) was carried out alone or in the presence of 200 µM of uric acid or 200 µM of ascorbic acid. In Figure 10, the percentage of DA peak current is reported in the different situations. The peak current is not modified in the presence of uric acid, showing no interference, whereas in the presence of ascorbic acid, an increase of 10% in the DA current peak is observed.

**Figure 11:**
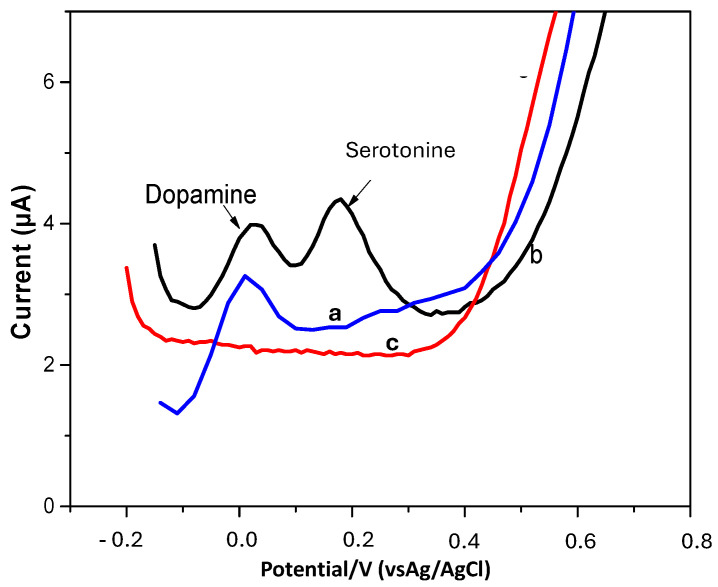
OCNF-modified SPCE (a), detection of 0.5 µM of dopamine at TOCNF-modified SPCE (0.01 M phosphate-buffered solution (pH 7.0)) (b), simultaneous detection of 0.5 µM serotonin and 0.5 µM dopamine at TOCNF-modified SPCE (c). Experimental conditions: 0.01 M phosphate-buffered solution (pH 7.0), scan rate 50 mV/s.

The electrochemical response of 0.5 µM dopamine was tested in the presence of 0.5 µM serotonin (Figure 11). The separation of both peaks was 160 mV, which is equal to that obtained with synthetic nanomaterials such as perovskites [39].

### 3.7. Analysis of Real Biological Samples

The TOCNF-based SPCE sensor’s performance was conducted by detecting dopamine (DA) in human urine samples. The urine samples were collected from healthy volunteers with informed consent, in accordance with the Declaration of Helsinki. The samples were then 10-fold diluted with 0.1 M PBS at pH 7. Subsequently, the standard addition method was applied, where different concentrations of dopamine were spiked into the prepared urine samples. As shown in Table 2, the recoveries were in the range of 98−116%, suggesting good feasibility and reliability of the TOCNF-based SPCE sensor for the detection of DA in urine samples.

## 4. Conclusions

In this study, an electrochemical sensor based on TEMPO-oxidized nanofibrillated cellulose (TOCNF) extracted from marram grass (*Ammophilia arenaria*) was developed successfully for the quantification of dopamine. The results show that TOCNFs greatly improved the oxidation peak of dopamine. The TOCNF-modified SPCE exhibits a high sensitivity of 7.92 µM/µA, good stability, and a low detection limit of 0.01µM for dopamine detection. The transfer coefficient of dopamine on this disposable TOCNF-modified electrode was close to that of the TOCNF/graphene composite-modified GCE. Moreover, due to its remarkable performance, the prepared sensor was tested for the determination of dopamine in real urine samples, yielding satisfactory results. The electrode material preparation was straightforward and efficient. These findings demonstrate that the disposable TOCNF-modified SPCE sensor exhibits excellent analytical performance for dopamine detection and holds great potential for future applications in biological fluids.

## Figures and Tables

**Table 1:** Comparison of TOCNF/SPCE sensor with other reported CNF-based electrochemical sensors for determination of dopamine.

Electrode	Method	Detection Limit (nM)	Linear Range (µM)	Reference
Sulfated CNF/MWCNT-modified taC	DPV	65	0.05–100	[30]
TOCNF/graphene/AgNP-modified GCE	DPV	0.5	0.005–250	[32]
TOCNF-modified SPCE	DPV	10	0.01–100	This work

**Table 2:** Results from the determination of DA with the TOCNF-modified SPCE sensor in a real sample.

Sample	Added (µM)	Found (µM)	Recovery (%)
Urine	0.06	0.07 ± 0.03	116
	0.10	0.11 ± 0.02	100
	0.60	0.61 ± 0.01	101
	1	0.98 ± 0.01	98

## Data Availability

The data that support the findings of this study are available from the corresponding author up on reasonable request.
